# 3-[4-(Dimethyl­amino)phenyl]-1-(3-pyridyl)prop-2-en-1-one

**DOI:** 10.1107/S1600536809013956

**Published:** 2009-04-18

**Authors:** A. Jayarama, Seik Weng Ng

**Affiliations:** a Department of Physics, Mangalore Institute of Technology and Engineering, Badagamijar, Moodabidri 574 225, India; b Department of Chemistry, University of Malaya, 50603 Kuala Lumpur, Malaysia

## Abstract

The pyridyl and aryl rings in the title compound, C_16_H_16_N_2_O, which are located at the ends of the propenone unit, are inclined at an angle of 17.1 (1)° with respect to each other.

## Related literature

For 3-(4-chloro­phenyl)-1-(3-pyridyl)prop-2-en-1-one, which crystallizes in a non-centrosymmetric space group, see: Uchida *et al.* (1998 ▶). For the general synthesis by the Claisen–Schmidt condensation, see: Vogel (1999 ▶). For literature on related compounds exhibiting second-harmonic generation activity, see: Gu *et al.* (2008 ▶); Ravindra *et al.* (2008*a* ▶,*b* ▶).
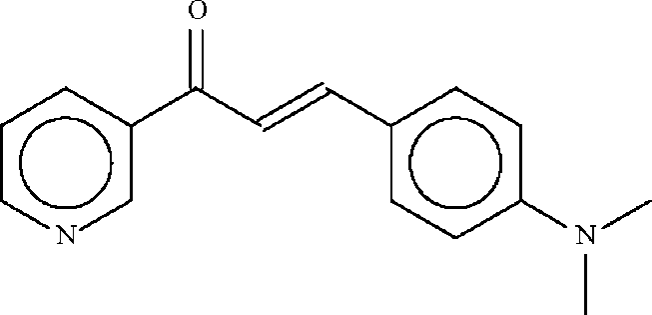


## Experimental

### 

#### Crystal data

C_16_H_16_N_2_O
*M* *_r_* = 252.31
Monoclinic, 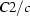

*a* = 14.6672 (6) Å
*b* = 11.0644 (4) Å
*c* = 16.7272 (6) Å
β = 107.205 (3)°
*V* = 2593.1 (2) Å^3^
*Z* = 8
Mo *K*α radiation
μ = 0.08 mm^−1^
*T* = 100 K
0.20 × 0.20 × 0.03 mm

#### Data collection

Bruker SMART APEX diffractometer
Absorption correction: none
11747 measured reflections
2976 independent reflections
1817 reflections with *I* > 2˘*I*)
*R*_int_ = 0.063

#### Refinement

*R*[*F* ^2^ > 2σ(*F* ^2^)] = 0.055
*wR*(*F* ^2^) = 0.147
*S* = 1.02
2976 reflections
174 parameters
H-atom parameters constrained
Δρ_max_ = 0.22 e Å^−3^
Δρ_min_ = −0.28 e Å^−3^

### 

Data collection: *APEX2* (Bruker, 2007 ▶); cell refinement: *SAINT* (Bruker, 2007 ▶); data reduction: *SAINT*; program(s) used to solve structure: *SHELXS97* (Sheldrick, 2008 ▶); program(s) used to refine structure: *SHELXL97* (Sheldrick, 2008 ▶); molecular graphics: *X-SEED* (Barbour, 2001 ▶); software used to prepare material for publication: *publCIF* (Westrip, 2009 ▶).

## Supplementary Material

Crystal structure: contains datablocks global, I. DOI: 10.1107/S1600536809013956/bt2931sup1.cif

Structure factors: contains datablocks I. DOI: 10.1107/S1600536809013956/bt2931Isup2.hkl

Additional supplementary materials:  crystallographic information; 3D view; checkCIF report

## Figures and Tables

**Fig. 1.**
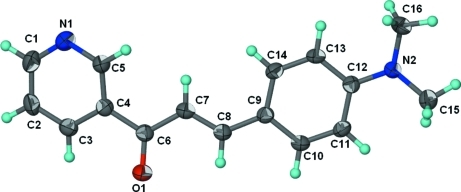
Anisotropic displacement ellipsoid plot (Barbour, 2001) of C16H16N2O at the 70% probability level. H atoms are drawn as spheres of arbitrary radius.

## supplementary crystallographic information

### Comment

Some chalcone derivatives exhibit high second-harmonic generation conversion efficiency (Gu *et al.*, 2008; Ravindra *et al.*, 2008*a*,*b*). The title compound was synthesized for the purpose of examining this property; unfortunately, the compound crystallizes in a centrosymmetric space group.

### Experimental

The compound was synthesized by the Claisen–Schmidt condensation (Vogel, 1999). To a mixture of ethanol (20 ml) and 10% sodium hydroxide solution (5 ml) was added an ethanol (15 ml) solution of 3-acetyl pyridine (0.001 mol) and 4-dimethylaminobenzaldehyde (0.001 mol). The temperature of the mixture was maintained at below 298 K for 2 h. The solid product that formed was washed with water. The compound was recrystallized from methanol.

### Refinement

H atoms were placed in calculated positions (C—H 0.95 to 0.98 Å) and were included in the refinement in the riding model approximation, with *U*(H) restrained to 1.2–1.5*U*_eq_(C).

### Crystal data

**Table:**

C_16_H_16_N_2_O	*F*(000) = 1072
*M**_r_* = 252.31	*D*_x_ = 1.293 Mg m^−^^3^
Monoclinic, *C*2/*c*	Mo *K*α radiation, λ = 0.71073 Å
Hall symbol: -C 2yc	Cell parameters from 1330 reflections
*a* = 14.6672 (6) Å	θ = 2.5–24.9°
*b* = 11.0644 (4) Å	µ = 0.08 mm^−^^1^
*c* = 16.7272 (6) Å	*T* = 100 K
β = 107.205 (3)°	Plate, orange
*V* = 2593.1 (2) Å^3^	0.20 × 0.20 × 0.03 mm
*Z* = 8	

### Data collection

**Table:**

Bruker SMART APEX diffractometer	1817 reflections with *I* > 2σ(*I*)
Radiation source: fine-focus sealed tube	*R*_int_ = 0.063
graphite	θ_max_ = 27.5°, θ_min_ = 2.4°
ω scans	*h* = −19→18
11747 measured reflections	*k* = −14→14
2976 independent reflections	*l* = −21→21

### Refinement

**Table:**

Refinement on *F*^2^	Primary atom site location: structure-invariant direct methods
Least-squares matrix: full	Secondary atom site location: difference Fourier map
*R*[*F*^2^ > 2σ(*F*^2^)] = 0.055	Hydrogen site location: inferred from neighbouring sites
*wR*(*F*^2^) = 0.147	H-atom parameters constrained
*S* = 1.02	*w* = 1/[σ^2^(*F*_o_^2^) + (0.0648*P*)^2^ + 0.8849*P*] where *P* = (*F*_o_^2^ + 2*F*_c_^2^)/3
2976 reflections	(Δ/σ)_max_ = 0.001
174 parameters	Δρ_max_ = 0.22 e Å^−^^3^
0 restraints	Δρ_min_ = −0.28 e Å^−^^3^

### Fractional atomic coordinates and isotropic or equivalent isotropic displacement parameters (Å^2^)

**Table:**

	*x*	*y*	*z*	*U*_iso_*/*U*_eq_	
O1	0.70214 (10)	0.23165 (12)	0.74962 (8)	0.0328 (4)	
N1	0.60015 (14)	0.57819 (16)	0.86721 (11)	0.0408 (5)	
N2	0.89331 (12)	0.58854 (14)	0.38128 (10)	0.0270 (4)	
C1	0.55057 (16)	0.5062 (2)	0.90340 (14)	0.0394 (6)	
H1	0.5185	0.5423	0.9390	0.047*	
C2	0.54329 (15)	0.3828 (2)	0.89203 (13)	0.0314 (5)	
H2	0.5073	0.3354	0.9192	0.038*	
C3	0.58905 (14)	0.33046 (18)	0.84076 (12)	0.0273 (5)	
H3	0.5855	0.2455	0.8322	0.033*	
C4	0.64088 (13)	0.40126 (16)	0.80103 (11)	0.0242 (4)	
C5	0.64452 (15)	0.52517 (17)	0.81678 (12)	0.0292 (5)	
H5	0.6802	0.5746	0.7905	0.035*	
C6	0.69040 (13)	0.34254 (16)	0.74515 (12)	0.0240 (4)	
C7	0.72162 (14)	0.41654 (17)	0.68607 (12)	0.0268 (5)	
H7	0.7108	0.5013	0.6846	0.032*	
C8	0.76531 (13)	0.36800 (17)	0.63362 (11)	0.0250 (4)	
H8	0.7750	0.2831	0.6380	0.030*	
C9	0.79945 (13)	0.42798 (16)	0.57141 (11)	0.0226 (4)	
C10	0.83957 (14)	0.35986 (17)	0.51975 (12)	0.0259 (5)	
H10	0.8451	0.2748	0.5277	0.031*	
C11	0.87122 (14)	0.41098 (16)	0.45818 (12)	0.0254 (4)	
H11	0.8973	0.3610	0.4243	0.030*	
C12	0.86545 (13)	0.53701 (16)	0.44457 (11)	0.0225 (4)	
C13	0.82739 (14)	0.60643 (17)	0.49786 (12)	0.0258 (5)	
H13	0.8240	0.6918	0.4916	0.031*	
C14	0.79515 (14)	0.55355 (17)	0.55848 (12)	0.0258 (4)	
H14	0.7692	0.6032	0.5927	0.031*	
C15	0.93708 (15)	0.51553 (18)	0.33043 (13)	0.0316 (5)	
H15A	0.8972	0.4445	0.3097	0.047*	
H15B	1.0006	0.4894	0.3644	0.047*	
H15C	0.9429	0.5635	0.2829	0.047*	
C16	0.91164 (16)	0.71857 (17)	0.38199 (13)	0.0332 (5)	
H16A	0.8521	0.7627	0.3761	0.050*	
H16B	0.9364	0.7392	0.3353	0.050*	
H16C	0.9587	0.7409	0.4350	0.050*	

### Atomic displacement parameters (Å^2^)

**Table:**

	*U*^11^	*U*^22^	*U*^33^	*U*^12^	*U*^13^	*U*^23^
O1	0.0461 (9)	0.0199 (7)	0.0374 (8)	−0.0033 (6)	0.0201 (7)	−0.0016 (6)
N1	0.0568 (13)	0.0308 (10)	0.0438 (11)	0.0060 (9)	0.0286 (10)	0.0004 (8)
N2	0.0349 (10)	0.0210 (8)	0.0292 (9)	0.0003 (7)	0.0159 (8)	0.0011 (7)
C1	0.0485 (15)	0.0406 (13)	0.0372 (13)	0.0114 (11)	0.0252 (12)	0.0032 (10)
C2	0.0320 (12)	0.0370 (12)	0.0291 (11)	−0.0004 (9)	0.0152 (9)	0.0073 (9)
C3	0.0316 (11)	0.0232 (10)	0.0268 (11)	−0.0005 (9)	0.0083 (9)	0.0011 (8)
C4	0.0254 (10)	0.0241 (10)	0.0228 (10)	0.0011 (8)	0.0069 (8)	0.0009 (8)
C5	0.0373 (12)	0.0242 (10)	0.0307 (11)	0.0013 (9)	0.0170 (10)	0.0012 (8)
C6	0.0268 (11)	0.0210 (10)	0.0245 (10)	−0.0036 (8)	0.0079 (9)	−0.0016 (8)
C7	0.0311 (11)	0.0207 (10)	0.0295 (11)	0.0012 (8)	0.0105 (9)	0.0016 (8)
C8	0.0302 (11)	0.0196 (9)	0.0262 (10)	−0.0024 (8)	0.0099 (9)	−0.0016 (8)
C9	0.0231 (10)	0.0207 (9)	0.0248 (10)	−0.0010 (8)	0.0084 (8)	−0.0013 (8)
C10	0.0318 (11)	0.0179 (9)	0.0289 (11)	−0.0004 (8)	0.0106 (9)	−0.0005 (8)
C11	0.0292 (11)	0.0211 (10)	0.0281 (10)	0.0001 (8)	0.0119 (9)	−0.0046 (8)
C12	0.0216 (10)	0.0226 (10)	0.0231 (10)	−0.0014 (8)	0.0065 (8)	−0.0003 (8)
C13	0.0315 (11)	0.0163 (9)	0.0311 (11)	0.0000 (8)	0.0115 (9)	−0.0004 (8)
C14	0.0285 (11)	0.0216 (9)	0.0302 (11)	0.0024 (8)	0.0130 (9)	−0.0023 (8)
C15	0.0349 (12)	0.0307 (11)	0.0335 (11)	−0.0019 (9)	0.0164 (10)	0.0003 (9)
C16	0.0405 (13)	0.0233 (10)	0.0394 (12)	−0.0023 (9)	0.0174 (10)	0.0045 (9)

### Geometric parameters (Å, °)

**Table:**

O1—C6	1.238 (2)	C8—C9	1.443 (2)
N1—C1	1.338 (3)	C8—H8	0.9500
N1—C5	1.343 (2)	C9—C10	1.401 (2)
N2—C12	1.367 (2)	C9—C14	1.405 (3)
N2—C15	1.453 (2)	C10—C11	1.371 (3)
N2—C16	1.463 (2)	C10—H10	0.9500
C1—C2	1.378 (3)	C11—C12	1.411 (2)
C1—H1	0.9500	C11—H11	0.9500
C2—C3	1.365 (3)	C12—C13	1.411 (3)
C2—H2	0.9500	C13—C14	1.370 (3)
C3—C4	1.390 (3)	C13—H13	0.9500
C3—H3	0.9500	C14—H14	0.9500
C4—C5	1.394 (3)	C15—H15A	0.9800
C4—C6	1.492 (2)	C15—H15B	0.9800
C5—H5	0.9500	C15—H15C	0.9800
C6—C7	1.458 (3)	C16—H16A	0.9800
C7—C8	1.342 (2)	C16—H16B	0.9800
C7—H7	0.9500	C16—H16C	0.9800
			
C1—N1—C5	116.96 (18)	C10—C9—C8	119.74 (17)
C12—N2—C15	120.43 (15)	C14—C9—C8	123.82 (17)
C12—N2—C16	120.01 (16)	C11—C10—C9	122.61 (17)
C15—N2—C16	116.07 (16)	C11—C10—H10	118.7
N1—C1—C2	123.9 (2)	C9—C10—H10	118.7
N1—C1—H1	118.1	C10—C11—C12	120.76 (18)
C2—C1—H1	118.1	C10—C11—H11	119.6
C3—C2—C1	118.4 (2)	C12—C11—H11	119.6
C3—C2—H2	120.8	N2—C12—C13	121.80 (16)
C1—C2—H2	120.8	N2—C12—C11	121.30 (17)
C2—C3—C4	120.10 (18)	C13—C12—C11	116.87 (17)
C2—C3—H3	120.0	C14—C13—C12	121.51 (17)
C4—C3—H3	120.0	C14—C13—H13	119.2
C3—C4—C5	117.36 (18)	C12—C13—H13	119.2
C3—C4—C6	119.39 (17)	C13—C14—C9	121.77 (18)
C5—C4—C6	123.24 (17)	C13—C14—H14	119.1
N1—C5—C4	123.34 (19)	C9—C14—H14	119.1
N1—C5—H5	118.3	N2—C15—H15A	109.5
C4—C5—H5	118.3	N2—C15—H15B	109.5
O1—C6—C7	122.12 (17)	H15A—C15—H15B	109.5
O1—C6—C4	118.59 (17)	N2—C15—H15C	109.5
C7—C6—C4	119.28 (16)	H15A—C15—H15C	109.5
C8—C7—C6	121.66 (17)	H15B—C15—H15C	109.5
C8—C7—H7	119.2	N2—C16—H16A	109.5
C6—C7—H7	119.2	N2—C16—H16B	109.5
C7—C8—C9	128.46 (18)	H16A—C16—H16B	109.5
C7—C8—H8	115.8	N2—C16—H16C	109.5
C9—C8—H8	115.8	H16A—C16—H16C	109.5
C10—C9—C14	116.44 (17)	H16B—C16—H16C	109.5
			
C5—N1—C1—C2	0.3 (3)	C7—C8—C9—C14	2.9 (3)
N1—C1—C2—C3	−0.1 (3)	C14—C9—C10—C11	−1.5 (3)
C1—C2—C3—C4	−0.4 (3)	C8—C9—C10—C11	178.42 (18)
C2—C3—C4—C5	0.8 (3)	C9—C10—C11—C12	0.6 (3)
C2—C3—C4—C6	−179.95 (18)	C15—N2—C12—C13	176.60 (17)
C1—N1—C5—C4	0.1 (3)	C16—N2—C12—C13	18.5 (3)
C3—C4—C5—N1	−0.6 (3)	C15—N2—C12—C11	−5.1 (3)
C6—C4—C5—N1	−179.85 (19)	C16—N2—C12—C11	−163.19 (18)
C3—C4—C6—O1	−15.8 (3)	C10—C11—C12—N2	−177.40 (18)
C5—C4—C6—O1	163.44 (19)	C10—C11—C12—C13	1.0 (3)
C3—C4—C6—C7	162.98 (18)	N2—C12—C13—C14	176.66 (18)
C5—C4—C6—C7	−17.8 (3)	C11—C12—C13—C14	−1.7 (3)
O1—C6—C7—C8	−0.4 (3)	C12—C13—C14—C9	0.8 (3)
C4—C6—C7—C8	−179.08 (18)	C10—C9—C14—C13	0.8 (3)
C6—C7—C8—C9	179.34 (18)	C8—C9—C14—C13	−179.16 (18)
C7—C8—C9—C10	−177.03 (19)		
